# Incarcerated Trocar Site Herniation of the Small Bowel following Laparoscopic Myomectomy

**DOI:** 10.1155/2013/202743

**Published:** 2013-07-14

**Authors:** William Kondo, Monica Tessmann Zomer, Andresa Braga-Baiak, Rafael Menezes de Azevedo

**Affiliations:** ^1^Department of Gynecology, Sugisawa Medical Center, Avenida Getulio Vargas, 3163 ap 21, 80240-041 Curitiba, PR, Brazil; ^2^Department of Radiology, Sugisawa Medical Center, Curitiba, PR, Brazil

## Abstract

Small bowel herniation through the fascial defect created by the entry of trocars is one of the major complications of the laparoscopic surgery. In this paper, we describe a 42-year-old woman developing an incarcerated trocar site herniation of the small bowel following laparoscopic myomectomy and treated by laparoscopic approach.

## 1. Introduction

Bowel herniation through the fascial defect created by the entry of trocars is one of the major complications of the laparoscopic surgery [1]. It may become a serious entity whenever incarceration of the port site herniation leads to bowel obstruction. In this paper, we describe a case of trocar site hernia presenting as an intestinal obstruction that was successfully treated by laparoscopic approach. 

## 2. Case Presentation

A 42-year-old woman, gravida 1 para 1, was referred to our service for a laparoscopic myomectomy. She complained about pelvic discomfort but her menses were normal. She did not want to undergo total hysterectomy because she desired pregnancy. Her body mass index was 31 kg/m². Transvaginal ultrasound showed an enlarged uterus (472 cm³) with a 91 × 68 × 75 mm subserous/intramural fibroid located at the right anterior uterine wall. 

She underwent laparoscopic myomectomy with transient uterine artery occlusion and extraction of the specimen by means of laparoscopic morcellation. The 15 mm morcellator was placed in the left iliac fossa and, as usual, the aponeurosis was closed using 1 polyglactin 910 at the end of the procedure (both the 10 mm umbilical incision and the 15 mm incision). The procedure, lasting 100 minutes, was uneventful, and the intraoperative bleeding was 50 mL. She was discharged home 28 hours after the procedure in good conditions. 

**Figure 1:**
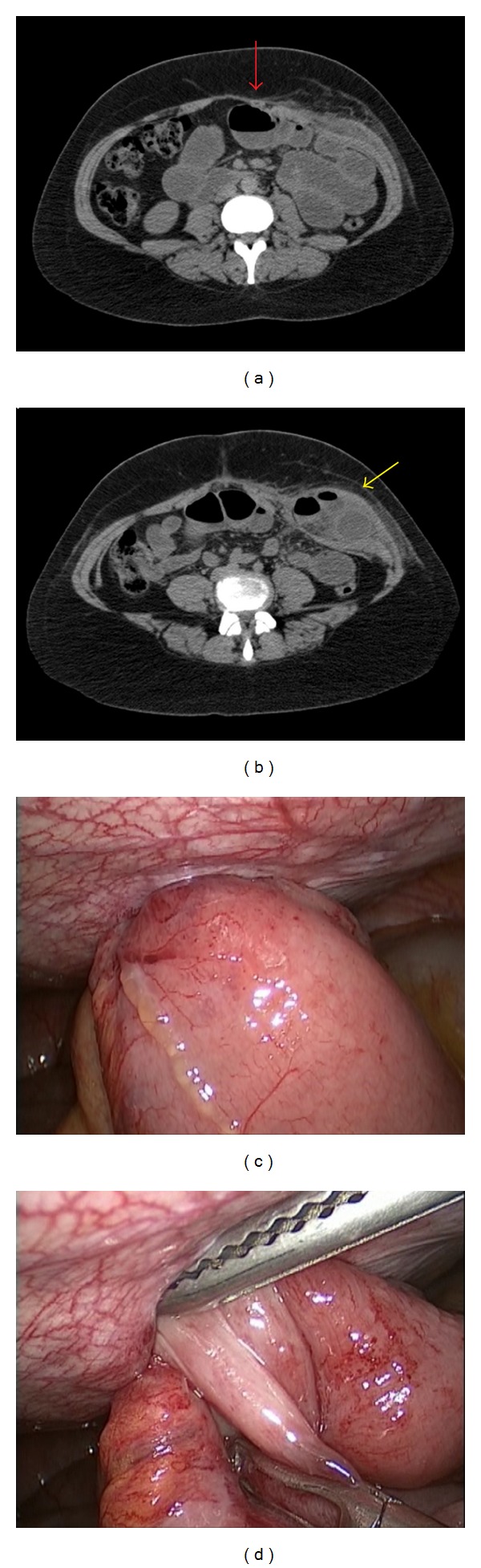
(a and b) Computed tomography of the abdomen and pelvis demonstrating the presence of small bowel loops herniated through the abdominal wall at the left iliac fossa (yellow arrow), with dilated proximal small bowel segments (red arrow), suggestive of incarcerated incisional hernia. (c and d) Laparoscopic surgery confirming the presence of an incarcerated herniation of the small bowel.

At home, she presented some episodes of epigastric burning pain, as well as nausea and vomiting, eventually. In the seventh postoperative day, she came back to the hospital complaining about vomiting and mild abdominal pain. On physical examination, she presented a palpable mass at the left iliac fossa (under the incision of the morcellator) with no flogistic signs. Laboratory investigation demonstrated hemoglobin of 13.4 g/dL, white blood cell count of 9,947/mm³ (band neutrophils 16%), and C-reactive protein of 133 mg/L. Computed tomography of the abdomen and pelvis was suggestive of incarcerated incisional hernia (Figures 1(a) and 1(b)). 

 She underwent another laparoscopic surgical procedure, with the identification of herniation of the small bowel through the trocar incision at the left iliac fossa along with incarceration (Figures 1(c) and 1(d)). The skin incision at the iliac fossa was opened (15 mm) and enlarged (10 mm), and the integrity of the aponeurosis was confirmed. So, the aponeurosis was opened, and the small bowel loops could be seen within the muscular layer of the abdominal wall. The parietal peritoneum and the abdominal wall muscles were opened laparoscopically, and the 20 cm of incarcerated and herniated small bowel loops were repositioned into the abdominal cavity. The viability of the bowel was confirmed (colour, vessels in mesentery, and peristalsis) after some minutes of observation. The muscular layer and the peritoneum were sutured using zero poliglecaprone 25 by laparoscopy, and the aponeurosis was closed using 1 poliglactin 910. 

 The patient presented a satisfactory postoperative course. Clear liquids were offered in the first postoperative day. A significative reduction of the levels of white blood cells and C-reactive protein was progressively observed, and the patient was discharged home in the fourth postoperative day. 

## 3. Discussion

Trocar-site hernia is defined by the development of a hernia at the cannula insertion site [2]. The larger the wound created by the trocar, the greater the risk of trocar site herniation. The incidence of postoperative trocar site hernia is estimated to be around 0.23% for 10 mm trocars, rising to 3.1% for 12 mm trocars [3]. 

 Depending on the time of presentation from the index operation, trocar-site hernias may be classified into three different types [4]. The early onset type usually develops within two weeks, has dehiscence of the fascial plane and the peritoneum, and most commonly presents with small bowel obstruction. The late onset type usually occurs after two weeks and has dehiscence of the fascial plane, but the peritoneum is intact and constitutes the hernial sac. The third type includes special types of hernia which have dehiscence of the whole abdominal wall with protrusion of the intestine and/or omentum.

 The clinical presentation of trocar-site hernias is variable and depends on the extent and nature of the herniated content [4, 5]. Swelling and pain at the incision site may be hard to differentiate from a postoperative hematoma or wound infection [6].

 The diagnosis of small bowel obstruction is made commonly on plain X-ray film. Ultrasound evaluation may be confusing in the postoperative period and is less accurate in the presence of an obese patient. CT scan is a useful tool to differentiate trocar-site hematoma from incarcerated small bowel and ileus from small bowel obstruction [7, 8].

 The management of trocar site herniation includes access to the hernia by extending the trocar-site incision, laparoscopy, or an explorative laparotomy and then reduction of the hernia and further surgeries based on the bowel viability [4].

 In order to avoid such a complication, it is recommended to close the fascial defect if the trocar size is larger than 10 mm [1, 9], especially in the presence of any risk factor for trocar-site herniation: (1) enlargement of the port-site incision to specimen retraction, (2) glucose intolerance, (3) obesity, (4) prolonged surgical time with extensive manipulation of the trocar (what may enlarge the trocar site), and (5) poor nutrition. The closure of the abdominal wall should preferentially include the peritoneum, as there are some literature reports, including ours, describing port-site herniation despite the closure of the superficial layer of the fascial defect [9, 10].

## 4. Conclusion

Although rare, trocar-site herniation is a possible complication of any laparoscopic procedure that should be kept in mind especially in those patients with signs and symptoms of small bowel obstruction in the early postoperative course. The early diagnosis and management are important to avoid further patient morbidity.
